# Diagnostic value of multimodal ultrasound for breast cancer and prediction of sentinel lymph node metastases

**DOI:** 10.3389/fcell.2024.1431883

**Published:** 2024-09-05

**Authors:** Hui Li, Lixia Chen, Meikuai Liu, Meng Bao, Quanbo Zhang, Shihao Xu

**Affiliations:** ^1^ Department of Ultrasound Imaging, The First Affiliated Hospital of Wenzhou Medical University, New District of the First Affiliated Hospital of Wenzhou Medical University, Wenzhou City, China; ^2^ Department of Pathology, The First Affiliated Hospital of Wenzhou Medical University, New District of the First Affiliated Hospital of Wenzhou Medical University, Wenzhou City, China

**Keywords:** breast cancer, multimodal ultrasound, lymph node metastases, LASSO regression, shear wave elastography, contrast-enhanced ultrasound

## Abstract

**Background:**

Sentinel lymph node metastasis (SLNM) is a critical factor in the prognosis and treatment planning for breast cancer (BC), as it indicates the potential spread of cancer to other parts of the body. The accurate prediction and diagnosis of SLNM are essential for improving clinical outcomes and guiding treatment decisions.

**Objective:**

This study aimed to construct a Lasso regression model by integrating multimodal ultrasound (US) techniques, including US, shear wave elastography (SWE), and contrast-enhanced ultrasound (CEUS), to improve the predictive accuracy of sentinel lymph node metastasis in breast cancer and provide more precise guidance for clinical treatment.

**Results:**

A total of 253 eligible samples were screened, of which 148 were group benign and 105 were group malignant. There were statistically significant differences (*p* < 0.05) between group malignant patients in terms of age, palpable mass, body mass index, distance to nipple, maximum diameter, blood flow, microcalcification, 2D border, 2D morphology, and 2D uniformity and group benign. The Lasso regression model was useful in the diagnosis of benign and malignant nodules with an AUC of 0.966 and in diagnosing SLNM with an AUC of 0.832.

**Conclusion:**

In this study, we successfully constructed and validated a Lasso regression model based on the multimodal ultrasound technique for predicting whether SLNM occurs in BCs, showing high diagnostic accuracy.

## Introduction

Data from 2020 show that, for the first time, the number of new cases of breast cancer (BC) at 2.26 million surpassed lung cancer globally, making it the most prevalent type of cancer, as well as topping the female cancer mortality rate with 680,000 deaths (Breidenbach et al., 2022). This trend emphasizes the critical importance of early detection and definitive diagnosis to improve survival and quality of life for BC patients. Although molybdenum X-rays and ultrasound (US) have become the dominant diagnostic methods, they have their limitations and potential risks, such as the applicability of molybdenum X-rays in Asian women and the risk of ionizing radiation, as well as overdiagnosis due to the US (Wang et al., 2020). MRI, although more capable of identifying breast soft tissue, sometimes needs to be used in combination with other methods due to its lack of specificity, which may trigger unnecessary overtreatment (Radhakrishna et al., 2018).

The determination of sentinel lymph node (SLN) metastasis is particularly critical in prognostic assessment. Although the vast majority of patients with early-stage BC show no clinically significant signs of lymph node metastasis, SLN metastases are still detected in 15%–20% of patients after surgery (Tonellotto et al., 2019). Current metastatic evaluation by lymph node dissection or biopsy, although routine, involves invasive means that are prone to complications and are not suitable for all patients. Therefore, there is an urgent need to develop a method for the non-invasive assessment of SLN status (Abass et al., 2018; Zhang et al., 2021).

Ultrasound elastography is commonly used as a diagnostic tool to help identify benign and malignant tumors, using color images to reflect tissue hardness. However, it carries the risks of mis- and underdiagnosis due to operator subjectivity (Zhao and Xu, 2019). To address this issue, the shear wave elastography (SWE) technique was developed. SWE quantitatively obtains Young’s modulus values, which allows for the objective evaluation of lesions and improves diagnostic reproducibility and accuracy (Chou et al., 2021). Additionally, contrast-enhanced ultrasound (CEUS) technology, a blood pool imaging technique formed by microbubble reflective interfaces, has shown significant advantages in displaying the microvasculature and has thus provided important information for invasive and metastatic studies of BC (Xu et al., 2020).

Multimodal ultrasound is an advanced medical imaging technology that combines different ultrasound imaging modalities to provide comprehensive information about a lesion, facilitating more accurate diagnosis and disease assessment (van der Pol et al., 2022). The advantages of multimodal ultrasound include significant reduction in the rate of misdiagnosis and omission and improving diagnostic efficiency and accuracy. As a non-invasive, portable, non-radioactive technology, it is particularly suitable for monitoring diseases that require frequent review (Zhu et al., 2020). This technique is widely used in the evaluation of soft tissue disorders such as thyroid nodules and liver disease.

In this study, multimodal ultrasound technology was used to integrate CEUS, US, and SWE methods to provide a comprehensive assessment of breast lesions. This approach requires a great deal of work and expertise, and ultrasound is highly operator-dependent. To address its fit within the regular workflow, it is important to clarify that multimodal ultrasound is intended for use in specific clinical scenarios. It is particularly useful for patients who have an abnormal mammogram or those with a known diagnosis of breast cancer, rather than being used as a general screening tool for all patients. Through comparative analysis with post-surgical pathological findings, this study explored the performance of each imaging technique and its value in BC diagnosis. Additionally, we attempted to establish a prediction model for SLN metastasis (SLNM) through this integrated diagnostic approach, aiming to provide a more accurate guide for clinical treatment.

## Methods and materials

### Sample information

The data of patients who were examined at the First Hospital of Wenzhou Medical University with breast masses from January 2023 to December 2023 were retrospectively collected. The study was conducted with the approval of the Medical Ethics Committee of the First Affiliated Hospital of Wenzhou Medical University (No. KY2022-R169).

### Inclusion and exclusion criteria

The inclusion criteria are as follows: ① Over 18 years of age. ② UE, SWE, and CEUS were performed before surgery. ③ Pathological results are available for surgery. ④ Pathology after surgery can clarify the presence or absence of SLNM. ⑤ Patient’s clinical data are complete.

The exclusion criteria are as follows: ① Patients who had undergone breast implantation. ② Image acquisition is not standard. ③ Patients with contraindications to surgery. ④ History of radiotherapy. ⑤ With contraindications to contrast agents. All subjects understood the content of the study, voluntarily participated in the study, and signed the informed consent form.

### Determination of benignity and malignancy

Patients’ tissues were obtained by surgery or pathology puncture and sent to the pathology department for testing to confirm the diagnosis of the tissue. The pathology results were used as the final judgement criteria.

### Sample screening

We screened 253 cases of eligible samples according to the inclusion and exclusion criteria. The patients were divided into group benign (n = 148) and group malignant (n = 105) according to the pathological test results.

### Ultrasound Detection

For ultrasound examination of breast lesions, we used a Resona 8 ultrasound system (provided by Mindray Medical International, Shenzhen, China). The system is equipped with a variety of probes, ranging from 3–15 MHz for US and shear wave elastography (SWE), and 3–11 MHz for contrast ultrasound (CEUS). The contrast agent used was SonoVue (provided by Bracco Suisse SA). First, the location, size, echo pattern, margins, shape, calcification, and blood flow condition of the lesion were assessed in detail by US examination, and this information was recorded. Subsequently, the SWE mode was initiated, and the main part of the lesion was placed in the center of the sampling frame. While instructing the patient to hold his/her breath, the mass-speed dual dynamic mode of SWE was selected. Image quality was considered good when the image showed a homogeneous green background with no significant purple artifacts. On this basis, we switched to grayscale SWE velocity dual mode, where red represents hard tissue and blue represents soft. The lesion was carefully observed for hard edge features (i.e., the area surrounding the lesion was red or orange). The assessment parameters are detailed in Supplementary Table S1. We manually outlined the edges of the lesion and recorded for the lesion mean elastic modulus (Emean), maximum elastic modulus (Emax), minimum elastic modulus (Emin), and elastic modulus standard deviation (Esd). We also recorded the peripheral area 2 mm around the tumor for mean elastic modulus (Esmean), maximum elastic modulus (Esmax), minimum elastic modulus (Esmin), and elastic modulus standard deviation (Essd). For the lesions plus peripheral areas, we recorded minimum elastic modulus (Elsmin), maximum elastic modulus (Elsmax), minimum elastic modulus (Elsmin) (0–140 kPa), and the elastic modulus standard deviation (Elssd). After completing the above steps, we replaced the probe and activated the CEUS mode. We selected a section with irregular morphology or abundant blood flow while ensuring that the normal breast tissue surrounding the lesion was also in the field of view. While 4.8 mL of the contrast agent was injected intravenously and immediately followed by 5 mL of saline, the timing and storage function were activated, and the images were continuously observed and recorded in real time for 3 min. During this procedure, detailed observation of the CEUS features of the lesion and its surrounding normal breast tissue was made, including enhancement time, enhanced intensity, enhancement margin, enhancement morphology, enhancement distribution, enhancement direction, enhancement area, Crab-claw-like pattern, perfusion defect, and Ring-like enhancement. In addition, it was necessary to record such factors as base intensity (BI), arrival time (AT), time to peak (TTP), peak intensity (PI), ascending slope (AS), 1/2 descending time (DT/2), decay slope (DS), area under the curve (AUC), and mean transit time (MTT).

### Clinical data collection

Information about the patient was obtained through electronic medical records, outpatient review records, and ultrasound test records. Clinical information included age, palpable mass, and body mass index (BMI). US parameters include location of the tumor, distance to the nipple, maximum diameter, aspect ratio, blood flow, microcalcification, 2D border, 2D morphology, 2D uniformity, and periductal features. SWE parameters include hard edge sign, Emean, Emax, Emin, Esd, Esmean, Esmax, Esmin, Essd, Elsmean, Elsmax, Elsmin, Elssd. CEUS parameters include enhancement margin, enhancement morphology, enhancement distribution, enhanced intensity, enhancement direction, perfusion defect, ring-like enhancement, enhancement area, Crab-claw-like pattern, enhancement time, BI, AT, TTP, PI, AS, DT/2, DS, AUC, MTT.

### Observation indicators

1. Analyze the difference in clinical data, US, SWE, and CEUS between group benign and group malignant patients.
2. Analyze the difference in clinical data, US, SWE, and CEUS between SLNM and non-SLNM patients.
3. Determine the optimal cut-off value for measures with differences by using receiver operating characteristic (ROC) curves.
4. Construct predictive models for benign and malignant tumors as well as SLNM by lasso-logistics regression.

### Statistical analysis

Statistical analyses were performed using SPSS 26.0 software. Count data were expressed as numbers, and χ^2^ test was used to compare rates between groups. Measurement information was expressed as x ± s, and a t-test was used. The diagnostic value of the model was analyzed using the ROC curve. The best cut-off value was obtained by ROC analysis, and the corresponding sensitivity, specificity were calculated. Lasso regression was performed using the “glmnet” package in R software (4.3. 2) with family = “Binomial or Gaussian”, alpha = 1, nfolds = 10. *p*-value <0.05 was considered statistically significant.

## Results

### Comparison of clinical data with US data

Comparison found that age, palpable mass, BMI, distance to nipple, maximum diameter, blood flow, microcalcification, 2D border, 2D morphology and 2D uniformity in group malignant patients were statistically different compared to group benign patients (*p* < 0.05, Table 1).

**TABLE 1:** Comparison of clinical data, ultrasonography, and color Doppler blood-flow imaging data.

Variables		Benign (n = 148)	Malignant (n = 105)	t/Z/χ^2^	*p*
Age (years)		42.65 ± 10.34	51.34 ± 10.97	6.361	<0.001
Palpable mass					
	Present	33	64	38.823	<0.001
	None	115	41		
Nipple discharge					
	Present	32	25	0.168	0.681
	None	116	80		
BMI (kg/m^2^)		22.30 [20.70,23.87]	23.05 [21.33,24.84]	2.544	0.011
Location of the tumor					
	Left	76	50	0.342	0.559
	Right	72	55		
Distance to nipple					
	>20 mm	13	23	8.664	0.003
	≤20 mm	135	82		
Maximum diameter					
	≥20 mm	28	53	28.103	<0.001
	<20 mm	120	52		
Aspect ratio					
	≥1	25	21	0.399	0.528
	<1	123	84		
Blood flow					
	Abundant	39	58	21.681	<0.001
	Sparse	109	47		
Microcalcification					
	Present	39	70	40.712	<0.001
	None	109	35		
2D border					
	Unclear	31	57	30.097	<0.001
	Clear	117	48		
2D morphology					
	Irregular	93	91	17.584	<0.001
	Regular	55	14		
2D uniformity					
	Homogeneous	56	20	10.319	0.001
	Heterogeneous	92	85		
Periductal features					
	Present	60	46	0.270	0.604
	None	88	59		

### Comparison of SWE parameters in benign and malignant patients

By comparison, hard edge sign, Emean, Emax, Emin, Esd, Esmean, Esmax, Essd, Elsmean, Elsmax, and Elssd were found to be statistically different in group malignant patients compared to group benign patients (*p* < 0.05, Table 2).

**TABLE 2:** SWE signs and parameters.

Variables		Benign (n = 148)	Malignant (n = 105)	Z/χ^2^	*p*
Hard edge sign					
	Yes	35	80	68.392	<0.001
	No	113	25		
Emean (kPa)		30.73 [21.81,45.13]	45.15 [39.20,52.73]	6.251	<0.001
Emax (kPa)		63.81 [43.24,89.19]	107.72 [82.93,150.58]	7.572	<0.001
Emin (kPa)		14.96 [8.56,19.91]	17.02 [10.99,25.01]	2.284	0.022
Esd		8.21 [5.95,12.50]	13.31 [10.47,18.45]	6.196	<0.001
Esmean (kPa)		33.69 [22.83,48.02]	50.35 [42.38,57.06]	6.581	<0.001
Esmax (kPa)		70.19 [49.31,105.69]	126.97 [101.44,167.53]	7.611	<0.001
Esmin (kPa)		12.25 [6.58,18.49]	14.15 [7.44,18.92]	1.205	0.229
Essd		11.18 [8.18,16.68]	18.66 [14.66,25.65]	6.79	<0.001
Elsmean (kPa)		33.08 [22.60,46.62]	48.60 [41.70,54.65]	6.376	<0.001
Elsmax (kPa)		74.85 [50.06,106.30]	133.63 [104.61,173.00]	7.844	<0.001
Elsmin (kPa)		12.00 [6.43,17.58]	13.23 [7.20,18.92]	0.989	0.323
Elssd		10.92 [7.85,15.36]	16.86 [12.84,21.47]	6.518	<0.001

### Comparison of CEUS parameters in benign and malignant patients

Enhancement morphology, enhanced intensity, ring-like enhancement, crab-claw-like pattern, ring-like enhancement, enhancement time, BI, and AT were found to be statistically different from group malignant patients by comparison. TTP, PI, DS, AUC, and MTT were statistically different compared with group benign patients (*p* < 0.05, Table 3).

**TABLE 3:** CEUS signs and parameters.

Variables		Benign (n = 148)	Malignant (n = 105)	t/Z/χ^2^	*p*
Enhancement margin					
	Clear	71	38	3.477	0.062
	Unclear	77	67		
Enhancement morphology					
	Regular	75	30	12.362	<0.001
	Irregular	73	75		
Enhancement distribution					
	Homogeneous	51	31	0.683	0.409
	Heterogeneous	97	74		
Enhanced intensity					
	High	76	96	45.327	<0.001
	None/low/equal	72	9		
Enhancement direction					
	Centripetal	109	85	1.832	0.176
	Centrifugal	39	20		
Perfusion defect					
	Present	70	55	0.635	0.426
	None	78	50		
Ring-like enhancement					
	Present	9	1	4.256	0.039
	None	139	104		
Crab-claw-like pattern					
	Present	8	45	52.025	<0.001
	None	140	60		
Enhancement area					
	>	31	84	86.396	<0.001
	=	117	21		
Enhancement time					
	Early	48	59	14.205	<0.001
	On time	100	46		
BI (db)		1.94 [1.26,2.97]	2.68 [1.58,4.65]	3.061	0.002
AT (s)		8.87 [6.91,11.22]	7.87 [6.31,9.52]	−2.856	0.004
TTP (s)		17.06 [14.25,20.48]	15.44 [12.89,18.06]	−3.185	0.001
PI (db)		20.46 [14.68,28.70]	29.61 [25.62,33.54]	5.880	<0.001
AS		0.57 [0.31,0.73]	0.61 [0.45,0.78]	1.941	0.052
DT/2 (s)		89.51 ± 29.30	96.61 ± 29.07	1.908	0.058
DS		−0.11 [-0.15,-0.07]	−0.12 [-0.16,-0.10]	−2.131	0.033
AUC		943.66 [581.58,1619.55]	1736.51 [1138.51,2156.63]	5.704	<0.001
MTT		79.42 ± 29.09	90.00 ± 29.40	2.833	0.005

### Determination of the optimal cut-off value

**FIGURE 1:**
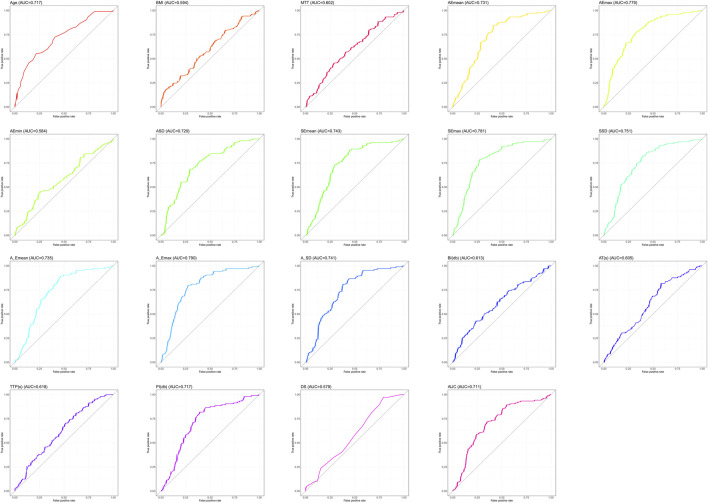
Multimodal ultrasound–parameter ROC curve.

Since Lasso-logistics regression requires the use of dichotomous data, we used ROC curves for the 19 measures that differed (Figure 1), and we subsequently calculated cut-off values for each measure (Table 4).

**TABLE 4:** ROC curve parameters.

Variables	AUC	95%CI	Specificity (%)	Sensitivity (%)	Youden index (%)	Cut_off
Age	0.717	0.653–0.780	78.38	55.24	33.62	50.5
BMI	0.594	0.523–0.665	34.46	80.00	14.46	21.13
Emean (kPa)	0.731	0.669–0.793	58.78	84.76	43.55	34.925
Emax (kPa)	0.779	0.722–0.837	66.89	78.10	44.99	79.855
Emin (kPa)	0.584	0.512–0.656	74.32	45.71	20.04	19.8
Esd	0.729	0.666–0.791	70.95	68.57	39.52	11.565
Esmean (kPa)	0.743	0.681–0.804	55.41	88.57	43.98	36.2
Esmax (kPa)	0.781	0.723–0.839	72.30	79.05	51.34	96.35
Essd	0.751	0.690–0.811	60.14	82.86	42.99	12.515
Elsmean (kPa)	0.735	0.673–0.797	53.38	89.52	42.90	34.235
Elsmax (kPa)	0.79	0.733–0.846	72.30	80.00	52.30	96.35
Elssd	0.741	0.680–0.802	62.16	80.95	43.11	12.29
BI	0.613	0.542–0.684	76.35	42.86	19.21	3.005
AT	0.605	0.536–0.675	41.89	81.90	23.80	10.065
TTP	0.618	0.549–0.687	48.65	70.48	19.12	17.445
PI	0.717	0.653–0.781	60.81	81.90	42.72	22.975
DS	0.579	0.509–0.649	20.95	97.14	18.09	−0.055
AUC	0.711	0.646–0.775	65.54	71.43	36.97	1291.22
MTT	0.602	0.532–0.673	71.62	45.71	17.34	93.745

### Lasso-logistics regression of benign and malignant tumor characteristic screening

**FIGURE 2:**
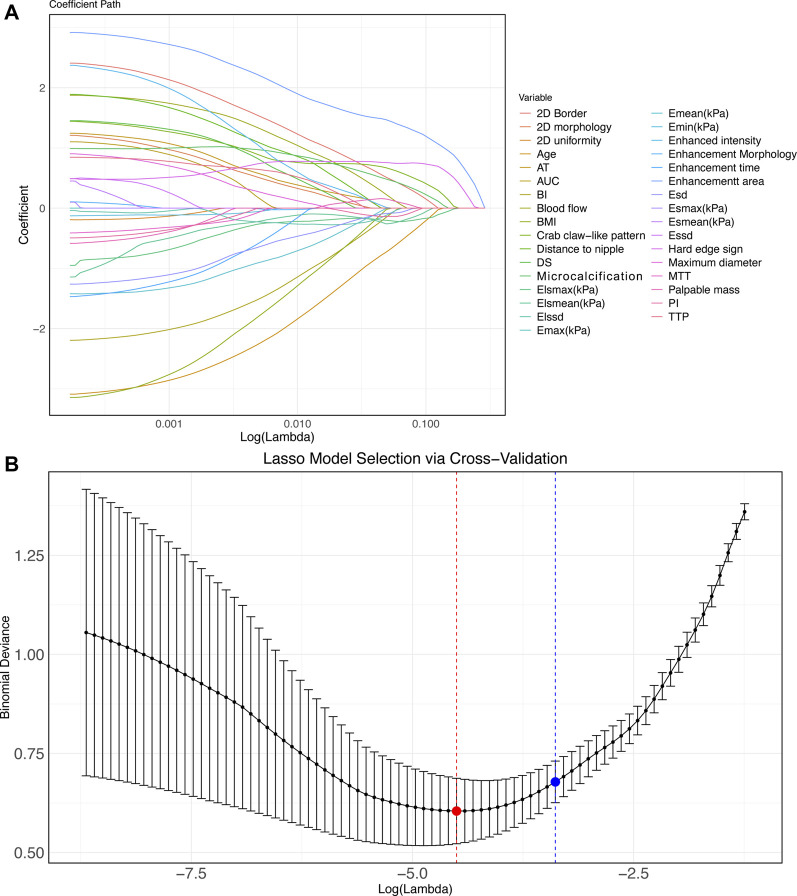
Lasso-logistics regression benign and malignant tumor feature screening **(A–B)** Characteristic variable screening with lambda values shown. Note: min points in red and 1se points in blue.

We assigned values to 33 meaningful variables (Supplementary Table S2). Subsequently, Lasso-logistics regression was used and two lambda values were filtered out, with 23 feature variables obtained when lambda = 0.0121 (min) and 21 feature variables obtained when lambda = 0.0306 (1se) (Figure 2). Considering the generalizability, we chose 21 feature variables at lambda = 0.0306 (1se) for model construction.

### Validation of Lasso-logistics regression model of benign and malignant tumors

**FIGURE 3:**
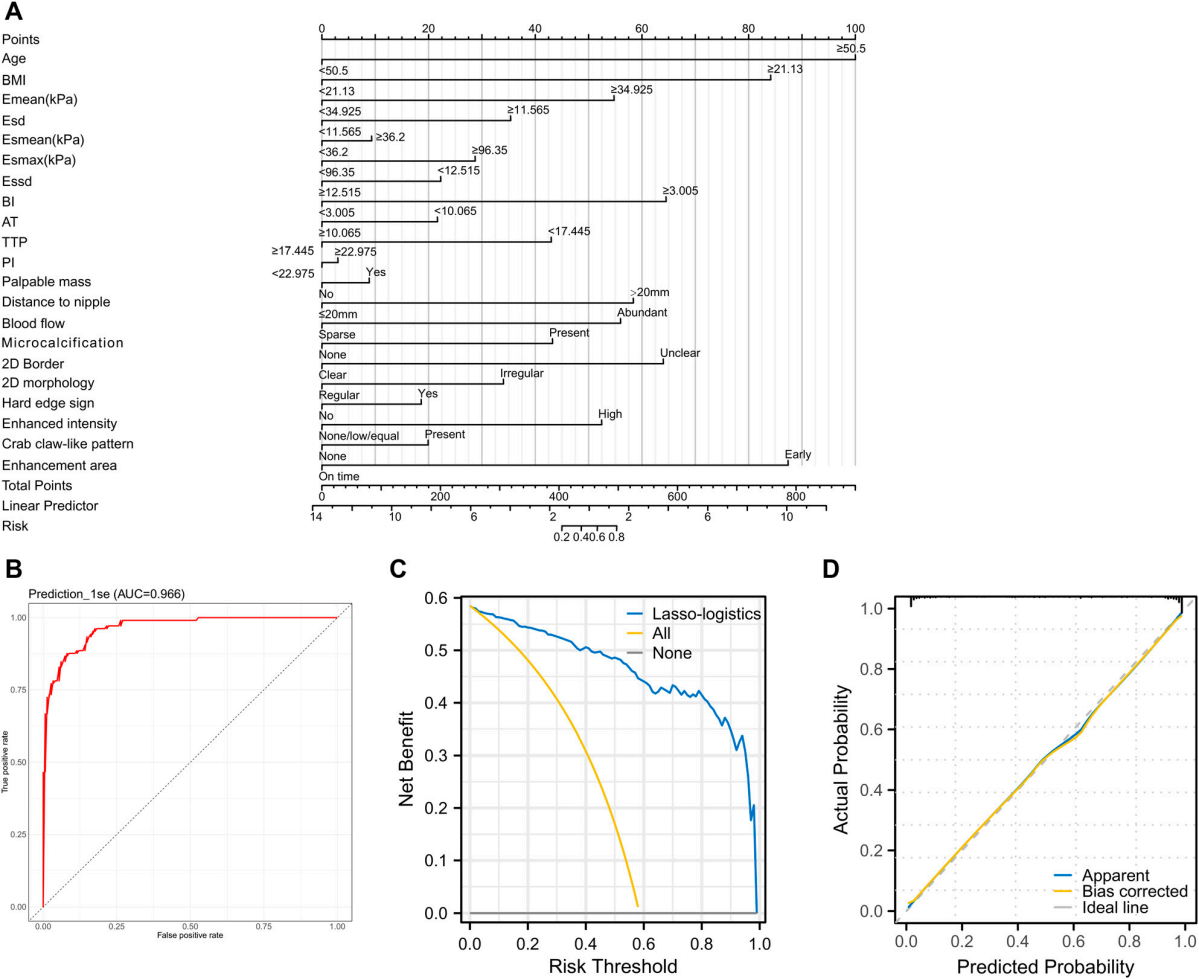
Validating the clinical value of the Lasso-logistics regression model **(A)** Nomogram plot of the 21 feature variables. **(B)** ROC curve to analyze the diagnostic value of the Lasso-logistics regression model. **(C)** DCA curve to analyze the clinical value of the Lasso-logistics regression model. **(D)** Calibration curves to analyze the accuracy of the Lasso-logistics regression model.

First, we constructed a nomogram (Figure 3A) based on 21 feature variables. We analyzed the value of the Lasso-logistics model in predicting benign and malignant tumors in patients using ROC curves, DCA curves, and calibration curves. The results found that the model had an AUC of 0.966 in diagnosing benign and malignant tumors (Figure 3B). In addition, DCA curve analysis found the model have a net yield of 58.49% (Figure 3C). The calibration curve analysis found the C-index of the model to be 0.966 (0.946–0.985), and the *p*-value of Hosmer–Lemeshow was 0.853 (Figure 3D). It suggested that the Lasso-logistics regression model has high value in diagnosing benign and malignant tumors.

### Lasso regression SLNM feature screening and model validation

**FIGURE 4:**
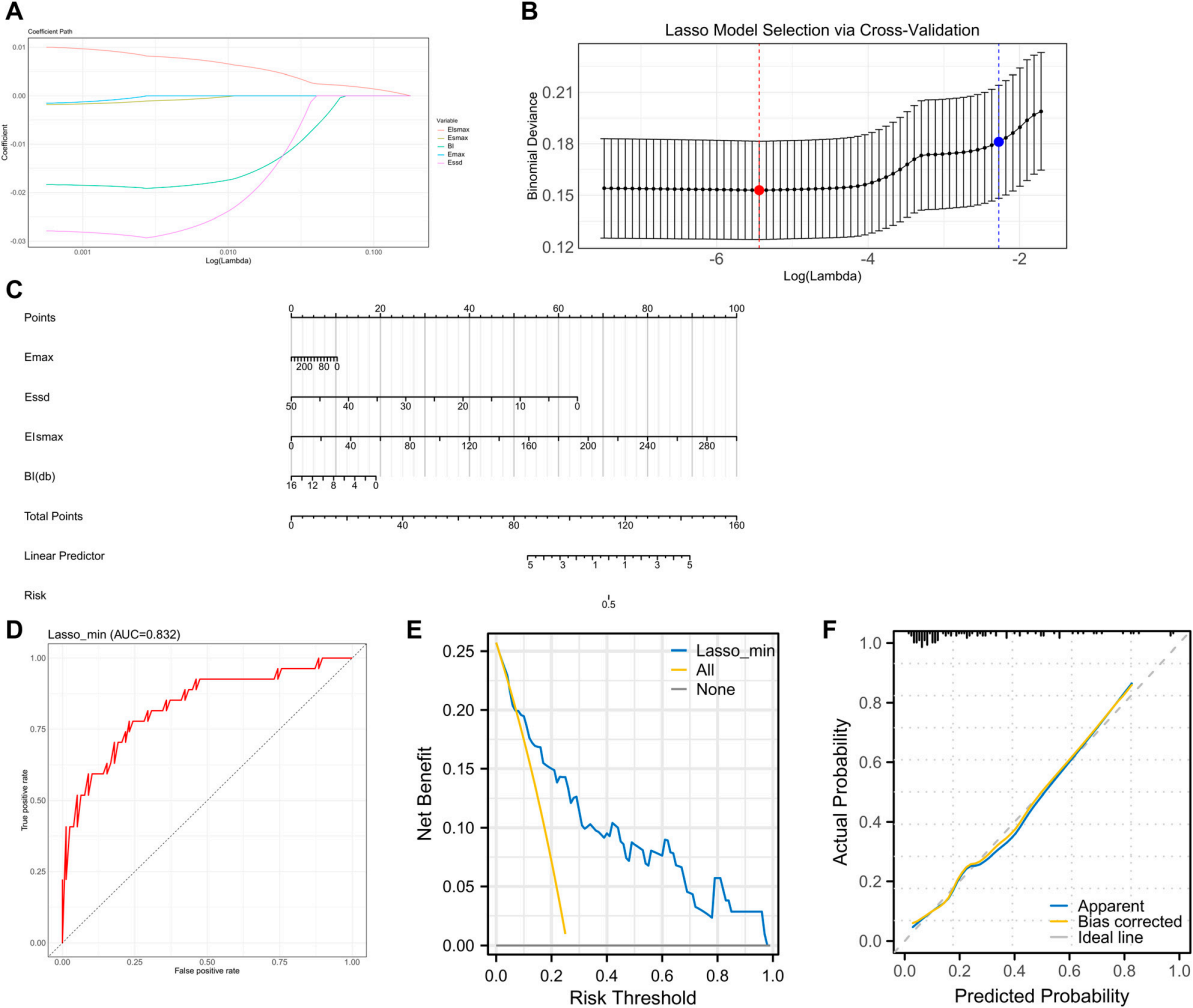
Lasso regression SLNM feature screening and model validation **(A–B)** Characteristic variable screening with lambda values shown, min points in red and 1se points in blue. **(C)** Nomogram plot of the four feature variables. **(D)** ROC curve analyzing the value of the Lasso regression model for diagnosing SLNM. **(E)** DCA curve analyzing the clinical value of the Lasso regression model for assessing SLNM. **(F)** Calibration curve to analyze the accuracy of the Lasso regression model for diagnosing SLNM.

We first grouped patients according to their metastases and subsequently compared all factors separately. The comparison revealed statistically significant differences in Elsmax, Esmax, Essd, Emax, and BI between metastasis group and non-metastasis group (*p* < 0.05, Tables 5, 6). As the initial screening revealed no differences in count information, we used the Gaussian function in Lasso regression for model construction. Four feature variables were acquired when lambda = 0.0043 (min), and one feature variable was acquired when lambda = 0.1027 (1se). Considering that choosing four feature variables (Emax, Essd, Elsmax, and BI) at lambda = 0.0043 (min) provides the best model accuracy and allows the model to capture the intrinsic relationships of the data more tightly, we decided to adopt this configuration for model construction (Figures 4A,B). The four feature variables were visualized by a nomogram (Figure 4C). ROC curve analysis revealed that the model had an AUC of 0.832 in diagnosing SLNM (Figure 4D). In addition, DCA curve analysis found that the model had a net yield of 25.71% (Figure E). The calibration curve analysis found the C-index of the model to be 0.832 (0.737–0.928), and the Hosmer–Lemeshow *p*-value was 0.749 (Figure F). This suggests that the Lasso regression model has some value in diagnosing SLNM.

**TABLE 5:** Comparison of ultrasound parameters and clinically relevant data measurements in patients with SLNM.

Variables	Metastasis group (n = 27)	Non-metastasis group (n = 78)	t/Z	*p*
Age	49.04 ± 11.50	52.14 ± 10.74	−1.229	0.226
BMI	23.30 [21.77,24.95]	22.66 [21.25,24.82]	0.66	0.512
Emean (kPa)	47.26 [43.50,50.34]	44.80 [37.56,53.89]	0.403	0.689
Emax (kPa)	131.82 [96.50,177.84]	98.94 [81.35,132.30]	4.245	<0.001
Emin (kPa)	16.18 [8.98,27.62]	17.02 [11.39,23.49]	−0.15	0.883
Esd	14.00 [10.95,17.55]	13.09 [10.29,18.67]	0.315	0.755
Esmean (kPa)	50.49 [47.70,61.41]	50.02 [41.80,56.16]	1.037	0.301
Esmax (kPa)	161.63 [125.05,202.56]	120.85 [96.83,146.42]	3.413	<0.001
Esmin (kPa)	8.79 [4.78,18.82]	15.29 [8.35,19.34]	−1.653	0.099
Essd	22.49 [16.87,28.01]	17.90 [13.01,22.35]	2.134	0.033
Elsmean (kPa)	49.10 [43.42,53.57]	47.91 [39.19,54.79]	0.579	0.565
Elsmax (kPa)	176.49 [145.67,210.97]	125.07 [96.83,150.38]	2.698	0.007
Elsmin (kPa)	8.79 [4.78,18.87]	13.77 [8.18,18.87]	−1.404	0.161
Elssd	18.41 [14.27,23.89]	15.25 [12.71,21.16]	1.602	0.11
BI (db)	2.28 [1.31,3.12]	2.75 [1.86,5.26]	−1.98	0.048
AT (s)	7.92 [6.36,9.16]	7.83 [6.24,9.50]	0.202	0.843
TTP (s)	15.19 [12.69,17.37]	15.50 [13.16,18.16]	−0.26	0.797
PI (db)	27.20 [23.41,33.39]	30.77 [25.66,33.54]	−1.213	0.226
AS	0.58 [0.44,0.80]	0.61 [0.48,0.77]	0.103	0.921
DT/2 (s)	88.11 ± 34.17	99.55 ± 26.71	−1.58	0.123
DS	−0.13 [-0.17,-0.10]	−0.12 [-0.15,-0.10]	−0.909	0.364
AUC	1554.03 ± 881.14	1760.84 ± 690.17	−1.108	0.275
MTT	79.75 ± 34.12	93.55 ± 26.92	−1.906	0.064

**TABLE 6:** Comparison of ultrasound parameters with clinically relevant information count data in patients with SLNM.

Variables		Metastasis group (n = 27)	Non-metastasis group (n = 78)	χ^2^	*p*
Palpable mass					
	Present	20	44	2.629	0.105
	None	7	34		
Nipple discharge					
	Present	4	21	1.621	0.203
	None	23	57		
Location of tumor					
	Left	13	37	0.004	0.949
	Right	14	41		
Distance to nipple					
	>20 mm	8	15	1.268	0.260
	≤20 mm	19	63		
Maximum diameter					
	≥20 mm	18	35	3.811	0.051
	<20 mm	9	43		
Aspect ratio					
	≥1	8	13	2.106	0.147
	<1	19	65		
Blood flow					
	Abundant	15	43	0.001	0.969
	Sparse	12	35		
Microcalcification					
	Present	21	49	2.019	0.155
	None	6	29		
2D border					
	Unclear	17	40	1.103	0.294
	Clear	10	38		
2D morphology					
	Irregular	25	66	1.105	0.293
	Regular	2	12		
2D uniformity					
	Homogeneous	3	17	1.485	0.223
	Heterogeneous	24	61		
Periductal features					
	Present	11	35	0.139	0.709
	None	16	43		
Hard edge sign					
	Yes	23	57	1.621	0.203
	No	4	21		
Enhancement margin					
	Clear	6	32	3.071	0.080
	Unclear	21	46		
Enhancement morphology					
	Regular	7	23	0.125	0.724
	Irregular	20	55		
Enhancement distribution					
	Homogeneous	6	25	0.931	0.335
	Heterogeneous	21	53		
Enhanced intensity					
	High	24	72	0.299	0.584
	None/low/equal	3	6		
Enhancement direction					
	Centripetal	23	62	0.422	0.516
	Non-centripetal	4	16		
Perfusion defect					
	Present	18	37	2.974	0.085
	None	9	41		
Ring-like enhancement					
	Present	1	0	2.917	0.088
	None	26	78		
Crab-claw-like pattern					
	Present	12	33	0.037	0.847
	None	15	45		
Enhancement area					
	>	23	61	0.611	0.435
	=	4	17		
Enhancement time					
	Early	12	47	2.037	0.154
	On time	15	31		

## Discussion

Early diagnosis of BC is essential to achieve timely treatment and improve patients’ quality of life (Mann et al., 2024). Through early detection and intervention, patients can choose more conservative treatment options such as surgical resection, radiotherapy, or endocrine therapy, thus avoiding more invasive treatments such as chemotherapy (Vicini et al., 2019). In addition, early diagnosis helps maintain the structural integrity of the breast, reduce tissue removal, lessen the postoperative physical form and emotional burden, and further enhance the patient’s quality of life (Loibl et al., 2024).

Although SWE has been utilized in breast imaging, it has not been widely adopted in clinical practice, mainly due to the high dependency on operator expertise and the significant cost of the equipment (He et al., 2023). However, SWE provides objective and reproducible diagnostic information by quantitatively measuring Young’s modulus of the tissues, which is crucial for improving the accuracy of breast cancer diagnosis (Chen et al., 2023). Previous studies have demonstrated the potential of SWE in differentiating benign and malignant breast lesions, yet its integration into routine clinical workflows remains limited (Weng and Yu, 2023). The combination of SWE with other imaging modalities, such as conventional US and CEUS, as explored in this study, may enhance its clinical utility and promote its adoption. By providing comprehensive and multimodal diagnostic information, this approach can potentially reduce the subjectivity associated with conventional US and offer a more reliable assessment of breast lesions and SLNM (Liu et al., 2019). Each of these techniques has limitations in the quantitative assessment of BC and may be missed or misdiagnosed when used alone. In the present study, we diagnosed benign and malignant tumors by multimodal ultrasound modality. In our study, we found that the multimodal ultrasound model constructed by lasso-logistics regression achieved an AUC of 0.966 in the diagnosis of BC, and we validated the clinical efficacy of the model by DCA curves and calibration curves. This result suggests that the combined use of multimodal ultrasound techniques with US, CEUS, and SWE can significantly improve the diagnostic accuracy of BC, especially in differentiating benign and malignant tumors. With this integrated approach, we are not only able to reduce the diagnostic errors that may be associated with a single technique, but also more accurately predict the nature of the tumor and thus provide more precise treatment recommendations for patients.

The multimodal ultrasound technique that combines US, CEUS, and SWE provides a highly accurate method for early cancer diagnosis and benign–malignant differentiation (Qian et al., 2021). Xiang et al. (2022) recently suggested that a fusion network constructed based on the three modalities of US, SWE, and CDUS effectively improves the diagnosis rate of thyroid nodules. They also indicated that multimodal ultrasound could enhance the diagnostic speed and efficiency of ultrasonographers. Additionally, Hu et al. (2020) had shown that the AUC of the ROC curve for diagnosing benign and malignant tumors of the liver using multimodal ultrasound techniques (US, CEUS, and SWE) was 0.968, much higher than individual ultrasound tests. Urhuț et al. (2023) demonstrated that the combined application of B-mode ultrasound, CEUS, pSWE, and RTE not only improved the diagnostic accuracy of hepatocellular carcinoma but also reduced the dependence on other imaging modalities. These results indicate that the multimodal ultrasound technology significantly enhances the diagnostic accuracy and efficiency in diagnosing diseases such as BC, thyroid nodules, and liver tumors. Each technique utilizes its unique strengths and complements the others to form a comprehensive assessment system, enhancing diagnostic accuracy, reducing misdiagnosis, improving efficiency, and reducing reliance on other imaging techniques (Han et al., 2022; Li et al., 2023).

Preoperative evaluation of SLNM in BC is critical for surgical decision-making and patient prognosis (Wang et al., 2022). Although 2D ultrasound is widely used in BC screening, it has limited sensitivity in identifying small or deep SLNM. Our study found significant differences in Elsmax, Esmax, Essd, Emax, and BI between patients with and without metastasis. Lasso regression analysis identified Emax, Essd, Elsmax, and BI as factors strongly associated with SLNM. Similarly, a study by Dai et al. (2022) proposed nomograms based on preoperative multimodal ultrasound features of papillary thyroid carcinoma and cervical lymph nodes to predict central lymph node metastasis. Their model demonstrated high predictive performance, especially in papillary thyroid carcinoma patients with varying tumor sizes. Furthermore, Dai et al. (2023) found that multimodal ultrasound has high clinical value in predicting central ultrasound radiomics models based lymph node metastasis in papillary thyroid carcinoma, with an AUC >0.9. These studies collectively confirm the effectiveness of combining multimodal ultrasound features and statistical models (e.g., Lasso regression and nomograms) in improving the accuracy of lymph node metastasis prediction in BC.

In this study, we successfully constructed a Lasso regression model based on the multimodal ultrasound technique to predict SLNM in BC. Although the model showed a high degree of predictive accuracy, we must acknowledge some of its limitations. First, the retrospective design of the study may have introduced selection and information biases, affecting our ability to generalize the results. Second, the relatively limited sample size of the study may not adequately represent the broader BC patient population. Third, although the model in this study performed well in preliminary tests, its long-term predictive effects and generalizability across different populations need to be further validated and explored. Future studies should use prospective designs, larger sample sizes, and multicenter collaborations to improve the generalizability and accuracy of predictive models. At the same time, more biomarkers and clinical information can be integrated to build comprehensive and refined models to facilitate personalized medicine and optimize treatment decisions.

## Conclusion

This study successfully constructed and validated two Lasso regression models based on the multimodal ultrasound technique for predicting BC and SLNM, showing high diagnostic accuracy.

## Data Availability

The original contributions presented in the study are included in the article/Supplementary Material; further inquiries can be directed to the corresponding author.

## References

[B1] Abass M. O. Gismalla M. D. A. Alsheikh A. A. Elhassan M. M. A. (2018). Axillary lymph node dissection for breast cancer: efficacy and complication in developing countries. J. Glob. Oncol. 4, 1–8. 10.1200/JGO.18.00080 PMC6223503 30281378

[B2] Breidenbach C. Heidkamp P. Hiltrop K. Pfaff H. Enders A. Ernstmann N. (2022). Prevalence and determinants of anxiety and depression in long-term breast cancer survivors. BMC Psychiatry 22 (1), 101. 10.1186/s12888-022-03735-3 35139815 PMC8827186

[B3] Chen X. Yu H. Wei N. Ozcan B. B. An G. Wu Q. (2023). Diagnostic performance of contrast-enhanced ultrasound combined with shear wave elastography in differentiating benign from malignant breast lesions: a systematic review and meta-analysis. Gland. Surg. 12 (11), 1610–1623. 10.21037/gs-23-333 38107493 PMC10721556

[B4] Chou S. S. Baikpour M. Zhang W. Mercaldo S. F. Lehman C. D. Samir A. E. (2021). Shear-wave elastography of the breast: impact of technical image quality parameters on diagnostic accuracy. AJR Am. J. Roentgenol. 216 (5), 1205–1215. 10.2214/AJR.19.22728 33729888

[B5] Dai Q. Liu D. Tao Y. Ding C. Li S. Zhao C. (2022). Nomograms based on preoperative multimodal ultrasound of papillary thyroid carcinoma for predicting central lymph node metastasis. Eur. Radiol. 32 (7), 4596–4608. 10.1007/s00330-022-08565-1 35226156

[B6] Dai Q. Tao Y. Liu D. Zhao C. Sui D. Xu J. (2023). Ultrasound radiomics models based on multimodal imaging feature fusion of papillary thyroid carcinoma for predicting central lymph node metastasis. Front. Oncol. 13, 1261080. 10.3389/fonc.2023.1261080 38023240 PMC10643192

[B7] Han Z. Huang Y. Wang H. Chu Z. (2022). Multimodal ultrasound imaging: a method to improve the accuracy of diagnosing thyroid TI-RADS 4 nodules. J. Clin. Ultrasound 50 (9), 1345–1352. 10.1002/jcu.23352 36169185

[B8] He H. Wu X. Jiang M. Xu Z. Zhang X. Pan J. (2023). Diagnostic accuracy of contrast-enhanced ultrasound synchronized with shear wave elastography in the differential diagnosis of benign and malignant breast lesions: a diagnostic test. Gland. Surg. 12 (1), 54–66. 10.21037/gs-22-684 36761482 PMC9906099

[B9] Hu J. Zhou Z. Y. Ran H. L. Yuan X. C. Zeng X. Zhang Z. Y. (2020). Diagnosis of liver tumors by multimodal ultrasound imaging. Med. Baltim. 99 (32), e21652. 10.1097/MD.0000000000021652 PMC7593067 32769936

[B10] Li G. Ma S. Zhang F. Jia C. Liu L. Gao F. (2023). The predictive models based on multimodality ultrasonography for the differential diagnosis of thyroid nodules smaller than 10 mm. Br. J. Radiol. 96 (1149), 20221120. 10.1259/bjr.20221120 37427752 PMC10461269

[B11] Liu G. Zhang M. K. He Y. Liu Y. Li X. R. Wang Z. L. (2019). BI-RADS 4 breast lesions: could multi-mode ultrasound be helpful for their diagnosis? Gland. Surg. 8 (3), 258–270. 10.21037/gs.2019.05.01 31328105 PMC6606479

[B12] Loibl S. André F. Bachelot T. Barrios C. H. Bergh J. Burstein H. J. (2024). Early breast cancer: ESMO Clinical Practice Guideline for diagnosis, treatment and follow-up. Ann. Oncol. 35 (2), 159–182. 10.1016/j.annonc.2023.11.016 38101773

[B13] Mann G. B. Skandarajah A. R. Zdenkowski N. Hughes J. Park A. Petrie D. (2024). Postoperative radiotherapy omission in selected patients with early breast cancer following preoperative breast MRI (PROSPECT): primary results of a prospective two-arm study. Lancet 403 (10423), 261–270. 10.1016/S0140-6736(23)02476-5 38065194

[B14] Qian X. Pei J. Zheng H. Xie X. Yan L. Zhang H. (2021). Prospective assessment of breast cancer risk from multimodal multiview ultrasound images via clinically applicable deep learning. Nat. Biomed. Eng. 5 (6), 522–532. 10.1038/s41551-021-00711-2 33875840

[B15] Radhakrishna S. Agarwal S. Parikh P. M. Kaur K. Panwar S. Sharma S. (2018). Role of magnetic resonance imaging in breast cancer management. South Asian J. cancer 7 (2), 69–71. 10.4103/sajc.sajc_104_18 29721466 PMC5909298

[B16] Tonellotto F. Bergmann A. de Souza Abrahão K. de Aguiar S. S. Bello M. A. Thuler L. C. S. (2019). Impact of number of positive lymph nodes and lymph node ratio on survival of women with node-positive breast cancer. Eur. J. breast health 15 (2), 76–84. 10.5152/ejbh.2019.4414 31001608 PMC6456272

[B17] Urhuț M. C. Săndulescu L. D. Ciocâlteu A. Cazacu S. M. Dănoiu S. (2023). The clinical value of multimodal ultrasound for the differential diagnosis of hepatocellular carcinoma from other liver tumors in relation to histopathology. Diagn. (Basel). 13 (20), 3288. 10.3390/diagnostics13203288 PMC10606610 37892109

[B18] van der Pol C. B. McInnes M. D. F. Salameh J. P. Levis B. Chernyak V. Sirlin C. B. (2022). CT/MRI and CEUS LI-RADS major features association with hepatocellular carcinoma: individual patient data meta-analysis. Radiology 302 (2), 326–335. 10.1148/radiol.2021211244 34783596

[B19] Vicini F. A. Cecchini R. S. White J. R. Arthur D. W. Julian T. B. Rabinovitch R. A. (2019). Long-term primary results of accelerated partial breast irradiation after breast-conserving surgery for early-stage breast cancer: a randomised, phase 3, equivalence trial. Lancet 394 (10215), 2155–2164. 10.1016/S0140-6736(19)32514-0 31813636 PMC7199428

[B20] Wang J. Zheng S. Ding L. Liang X. Wang Y. Greuter M. J. W. (2020). Is ultrasound an accurate alternative for mammography in breast cancer screening in an asian population? A meta-analysis. Diagn. (Basel) 10 (11), 985. 10.3390/diagnostics10110985 PMC7700617 33233479

[B21] Wang X. Zhang G. Zuo Z. Zhu Q. Wu S. Zhou Y. (2022). Sentinel lymph node positive rate predicts non-sentinel lymph node metastasis in breast cancer. J. Surg. Res. 271, 59–66. 10.1016/j.jss.2021.09.039 34839110

[B22] Weng L. Yu M. (2023). Diagnosis of benign and malignant BI-RADS 4 breast masses by contrastenhanced ultrasound combined with shear wave elastography. Curr. Med. imaging 20. 10.2174/0115734056257195231025072821 37921152

[B23] Xiang Z. Zhuo Q. Zhao C. Deng X. Zhu T. Wang T. (2022). Self-supervised multi-modal fusion network for multi-modal thyroid ultrasound image diagnosis. Comput. Biol. Med. 150, 106164. 10.1016/j.compbiomed.2022.106164 36240597

[B24] Xu P. Yang M. Liu Y. Li Y. P. Zhang H. Shao G. R. (2020). Breast non-mass-like lesions on contrast-enhanced ultrasonography: feature analysis, breast image reporting and data system classification assessment. World J. Clin. Cases 8 (4), 700–712. 10.12998/wjcc.v8.i4.700 32149054 PMC7052556

[B25] Zhang L. Jia Z. Leng X. Ma F. (2021). Artificial intelligence algorithm-based ultrasound image segmentation technology in the diagnosis of breast cancer axillary lymph node metastasis. J. Healthc. Eng. 2021, 8830260. 10.1155/2021/8830260 34367541 PMC8339348

[B26] Zhao C. K. Xu H. X. (2019). Ultrasound elastography of the thyroid: principles and current status. Ultrasonogr. Seoul. Korea. 38 (2), 106–124. 10.14366/usg.18037 PMC6443591 30690960

[B27] Zhu A. Q. Li X. L. An L. W. Guo L. H. Fu H. J. Sun L. P. (2020). Predicting axillary lymph node metastasis in patients with breast invasive ductal carcinoma with negative axillary ultrasound results using conventional ultrasound and contrast-enhanced ultrasound. J. Ultrasound Med. 39 (10), 2059–2070. 10.1002/jum.15314 32367518

