# Supplementary material for: Diagnostic value of multimodal ultrasound for breast cancer and prediction of sentinel lymph node metastases
Source: Front Cell Dev Biol. 2024 Sep 5;12:1431883. doi: 10.3389/fcell.2024.1431883 (PMC11411459; doi:10.3389/fcell.2024.1431883)
Supplement: Supplementary file 1 [file Table1.docx]

**Supplement Table 1** Comprehensive Ultrasound Elasticity and Contrast Enhancement Parameters for Breast Lesion Assessment

|  | **Features** | **Full Name** |
| --- | --- | --- |
| **Primary Lesion Assessment** (Manually outline the edges of the lesion and record the detailed characteristics of the lesion.) | Emean | Elastic Modulus Mean |
|  | Emax | Elastic Modulus Maximum |
|  | Emin | Elastic Modulus Minimum |
| **Peripheral Elasticity Assessment** (Evaluate the elasticity of the tissue surrounding the tumor within a 2mm range to assess tumor invasiveness and the reaction of adjacent tissues.) | Esmean | Elastic Modulus Mean in the Peripheral Region |
|  | Esmax | Elastic Modulus Maximum in the Peripheral Region |
|  | Esmin | Elastic Modulus Minimum in the Peripheral Region |
|  | Essd | Elastic Modulus Standard Deviation in the Peripheral Region |
| **Comprehensive Lesion and Periphery Analysis** (Integrate measurements from both the primary lesion and its surrounding area to provide a comprehensive assessment.) | Elsmin | Elastic Modulus Minimum in the Lesion plus Peripheral Region |
|  | Elsmax | Elastic Modulus Maximum in the Lesion plus Peripheral Region |
|  | Elssd | Elastic Modulus Standard Deviation in the Lesion plus Peripheral Region |
| **Contrast-Enhanced Ultrasound (CEUS) Imaging** (After the initial assessments, switch the probe to CEUS mode. Choose a section displaying irregular morphology or significant blood flow, ensuring inclusion of the surrounding normal breast tissue in the imaging field. Administer 4.8 ml of contrast agent intravenously, immediately followed by 5 ml of saline. Activate the timing and storage functions, and continuously observe and record the images in real-time over three minutes.) | Enhancement time | / |
|  | Enhanced intensity | / |
|  | Enhancement Margin | / |
|  | Enhancement Morphology | / |
|  | Enhancement Distribution (Uniformity) | / |
|  | Enhancement Direction | / |
|  | Enhancement Area (Expansion) | / |
|  | Crab-claw-like pattern | / |
|  | Perfusion Defect | / |
|  | Ring-like enhancement | / |
| **Others** | BI | Blood Intensity |
|  | AT | Arrival Time |
|  | TTP | Time to Peak |
|  | PI | Peak Intensity |
|  | AS | Acoustic Shadowing |
|  | DT/2 | Decay Half |
|  | DS | Decay Slope |
|  | AUC | Area Under the Curve |
|  | MTT | Mean Transit Time |
