# Supplementary material for: Diagnostic value of multimodal ultrasound for breast cancer and prediction of sentinel lymph node metastases
Source: Front Cell Dev Biol. 2024 Sep 5;12:1431883. doi: 10.3389/fcell.2024.1431883 (PMC11411459; doi:10.3389/fcell.2024.1431883)
Supplement: Supplementary file 2 [file Table2.docx]

**Supplementary Table 2 Variable Assignment Table**

| Variables | Assignment |
| --- | --- |
| Age | ＜50.5 = 0, ≥50.5 = 1 |
| BMI | ＜21.13 = 0, ≥21.13 = 1 |
| Emean(kPa) | ＜34.925=0, ≥34.925 = 1 |
| Emax(kPa) | ＜79.855 = 0, ≥79.855 = 1, |
| Emin(kPa) | ＜19.8 = 0, ≥19.8 = 1 |
| Esd | ＜11.565 = 0, ≥11.565 = 1 |
| Esmean(kPa) | ＜36.2 = 0, ≥36.2 = 1 |
| Esmax(kPa) | ＜96.35 = 0, ≥96.35 = 1 |
| Essd | ＜12.515 = 0, ≥12.515 = 1 |
| Elsmean(kPa) | ＜34.235 = 0, ≥34.235 = 1 |
| Elsmax(kPa) | ＜96.35 = 0, ≥96.35= 1 |
| Elssd | ＜12.29 = 0, ≥12.29=1 |
| BI | ＜3.005 = 0, ≥3.005=1 |
| AT | ≥10.065 = 0, ＜10.065=1 |
| TTP | ≥17.445 = 0, ＜17.445 = 1 |
| PI | ＜22.975 = 0, ≥22.975 = 1 |
| DS | ≥-0.055 = 0, ＜-0.055 = 1 |
| AUC | ＜1291.22 = 0, ≥1291.22 = 1 |
| MTT | ＜93.745 = 0, ≥93.745 = 1 |
| Palpable mass | None = 0, Present = 1 |
| Distance to nipple | ≤20mm = 0, ＞20mm = 1 |
| Maximum diameter | ≤20mm = 0, ＞20mm = 1 |
| Blood flow | Sparse = 0, Abundant = 1 |
| Echogenicity | None = 0, Present = 1 |
| 2D Border | Clear = 0, Unclear = 1 |
| 2D morphology | Regular = 0, Irregular = 1 |
| 2D uniformity | Nonuniform = 0, Uniform = 1 |
| Hard edge sign | None=0, Present = 1 |
| Enhancement Morphology | Regular = 0, Irregular = 1 |
| Enhanced intensity | None, Low, Equal = 0, High = 1 |
| Crab claw-like pattern | None = 0, Present = 1 |
| Enhancement area | Equal = 0, Enlarge = 1 |
| Enhancement time | No Earlier = 0, Earlier = 1 |
